# Evidence of non-*Plasmodium falciparum* malaria infection in Kédougou, Sénégal

**DOI:** 10.1186/s12936-016-1661-3

**Published:** 2017-01-03

**Authors:** Rachel F. Daniels, Awa Bineta Deme, Jules F. Gomis, Baba Dieye, Katelyn Durfee, Julie I. Thwing, Fatou B. Fall, Mady Ba, Medoune Ndiop, Aida S. Badiane, Yaye Die Ndiaye, Dyann F. Wirth, Sarah K. Volkman, Daouda Ndiaye

**Affiliations:** 1 Department of Immunology and Infectious Disease, Harvard T.H. Chan School of Public Health, Boston, MA USA; 2 Infectious Disease Initiative, Broad Institute of MIT and Harvard, Cambridge, MA USA; 3 Department of Parasitology and Mycology, Cheikh Anta Diop University, Dakar, Senegal; 4 Laboratory of Parasitology and Mycology, Cheikh Anta Diop University/Le Dantec Hospital, Dakar, Senegal; 5 Malaria Branch, Center for Global Health, Centers for Disease Control and Prevention, Atlanta, GA USA; 6 President’s Malaria Initiative, Dakar, Senegal; 7 National Malaria Control Programme, Dakar, Senegal; 8 School of Nursing and Health Sciences, Simmons College, Boston, MA USA

**Keywords:** *Plasmodium falciparum*, *Plasmodium ovale curtisi*, *Plasmodium ovale wallikeri*, *Plasmodium malariae*, Rapid diagnostic test

## Abstract

**Background:**

Expanded malaria control efforts in Sénégal have resulted in increased use of rapid diagnostic tests (RDT) to identify the primary disease-causing *Plasmodium* species, *Plasmodium falciparum*. However, the type of RDT utilized in Sénégal does not detect other malaria-causing species such as *Plasmodium ovale* spp., *Plasmodium malariae*, or *Plasmodium vivax*. Consequently, there is a lack of information about the frequency and types of malaria infections occurring in Sénégal. This study set out to better determine whether species other than *P. falciparum* were evident among patients evaluated for possible malaria infection in Kédougou, Sénégal.

**Methods:**

Real-time polymerase chain reaction speciation assays for *P. vivax, P. ovale* spp., and *P. malariae* were developed and validated by sequencing and DNA extracted from 475 *Plasmodium falciparum*-specific HRP2-based RDT collected between 2013 and 2014 from a facility-based sample of symptomatic patients from two health clinics in Kédougou, a hyper-endemic region in southeastern Sénégal, were analysed.

**Results:**

*Plasmodium malariae* (n = 3) and *P. ovale wallikeri* (n = 2) were observed as co-infections with *P. falciparum* among patients with positive RDT results (n = 187), including one patient positive for all three species. Among 288 negative RDT samples, samples positive for *P. falciparum* (n = 24), *P. ovale curtisi* (n = 3), *P. ovale wallikeri* (n = 1), and *P. malariae* (n = 3) were identified, corresponding to a non-*falciparum* positivity rate of 2.5%.

**Conclusions:**

These findings emphasize the limitations of the RDT used for malaria diagnosis and demonstrate that non-*P. falciparum* malaria infections occur in Sénégal. Current RDT used for routine clinical diagnosis do not necessarily provide an accurate reflection of malaria transmission in Kédougou, Sénégal, and more sensitive and specific methods are required for diagnosis and patient care, as well as surveillance and elimination activities. These findings have implications for other malaria endemic settings where species besides *P. falciparum* may be transmitted and overlooked by control or elimination activities.

**Electronic supplementary material:**

The online version of this article (doi:10.1186/s12936-016-1661-3) contains supplementary material, which is available to authorized users.

## Background

Among *Plasmodium* species that infect humans, *Plasmodium falciparum* and *Plasmodium vivax* are responsible for most of the global mortality and morbidity. However, with intensified efforts to control the incidence of these primary species, there is rising concern over the prevalence of additional malaria species including *Plasmodium ovale* spp. and *Plasmodium malariae* into the niches previously occupied by *P. falciparum* and *P. vivax* [1–3]. Should these other species be undetected by current diagnostic methods, there is the potential that they may make substantive and possibly increasing contributions to overall malaria burden.

Globally, there have been sporadic reports of changing trends in the prevalence of malaria species other than *P. falciparum*. For example, there are increasing reports of *P. ovale* spp. in various countries across the African continent, including Ethiopia, Uganda, Equatorial Guinea, and Kenya [1–3]. Historically, and recently, there have been *ad hoc* reports of malaria infections caused by species other than *P. falciparum* in Sénégal. While previous reports in Sénégal have indicated low or reduced incidence of these species [4, 5], there are limited data from other regions of the country, and it remains unknown if these observations can be generalized nationwide. Furthermore, *P. malariae* is relatively under-reported in these regions [6]. Recently, multiple clinical cases of *P. vivax* infection were detected in Sénégal [7]. However, it remains unclear if the paucity of data describing other malaria species is due to a lack of inadequate diagnostics or a consequence of relative lack of transmission in these regions. Overall there is insufficient information about the prevalence or dynamics of these malaria species, especially in the context of recent efforts to reduce or eliminate malaria. Moreover, no recent attempt has been made to systematically determine the prevalence of non-falciparum malaria parasites in Sénégal.

The region of Kédougou in southeastern Sénégal is an area of high malaria transmission that borders the countries of Mali and Guinea. It is characterized by relatively high humidity and an ecology that supports seasonal malaria transmission, with entomological inoculation rates between 100 and 200 and incidence rates of greater than 25 per 1000 individuals [8–10]. Human migration to and from the region is encouraged by ongoing mining activities in Kédougou that have expanded in the most recent decade.

In addition to using previously-reported methods, this study also developed and validated a set of real-time PCR speciation assays to detect *P. ovale* spp., *P. malariae,* and *P. vivax*. These assays were applied to analyse nearly 500 rapid diagnostic tests (RDT) from two health centers in Kédougou, Sénégal in order to assess the prevalence of non-*falciparum* species in this region.

## Methods

### Sample collection site

**Fig. 1:**
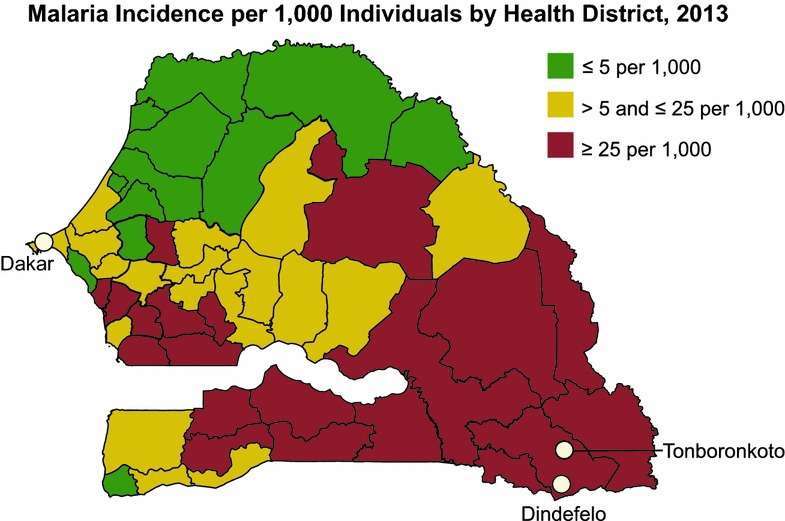
Study area. Map of Sénégal showing the location of health clinics in Kédougou relative to Dakar. *Shading* indicates malaria incidence levels. Figure adapted from the National Strategic Framework 2014–2018 [10]

Kédougou (12°56′00″ N, 12º21,00″W) is situated in the extreme southeastern part of Sénégal, in a tropical savanna located 710 km from Dakar, the nation’s capital (Fig. 1). Its 151,715 inhabitants comprise around 5% of the national population. The passive case detection in this study was based on patients aged 6 months or older reporting to health clinics in Tomboronkonto and Dindefelo for suspected malaria with fever or history of fever in the previous 48 h. Samples were collected from the first patient over 5 years of age as well as the first patient under 5 years of age reporting daily, for 4 days of the week over the course of a single year, a sampling scheme considered roughly representative of the symptomatic population reporting to health care facilities. These patients were diagnosed by RDT (First Response Malaria Ag [HRP2], Premier Medical Corp., USA), and parasite material from these discarded RDT was extracted for species identification. Institutional Review Board (IRB) review of these discarded diagnostic materials that were anonymous and de-identified were deemed non-human subjects material and used accordingly.

### Sample type and extraction

Parasite DNA was isolated from the filter pad of the RDT using the Promega DNA IQ Casework Pro Kit for Maxwell 16 (Promega Corp., Madison, WI, USA) as directed by the manufacturer.

### Speciation assays

First, species-specific TaqMan real-time assays were developed to target the *Plasmodium plasmepsin 4* gene based on previously-reported sequence data [11] (Table 1). Each DNA sample was run in duplicate with plasmid-based standard curves from 50,000 to 0.5 copies. The control plasmids contained species-specific *plasmepsin 4* genes (Genbank accession numbers AF001208.1, AF001209.1, and 144 AF001210.1 for *P. vivax, P. ovale* spp., and *P. malariae*, respectively).

**Table 1:** Primer and probe sequences

Gene	Primer name	Primer sequence (5′– >3′)	Amplicon size (bp)	Specificity	Reference
Porbp2	Porbp2TMfwd	TTGCAAACAAAAGTGCTCC	120	*P. ovale*	[2]
	Porbp2TMrev	CCTAATTCTCTTTGT(G/A)CCC			
Potra	PoTRA fwd3	GCACAAAAATGGTGCTAACC	787	*P. ovale*	[2]
	PoTRA rev3	ATCCATTTACCTTCCATTGC			
	PoTRA fwd5	ACGGCAAACCCGATAAACAC	245–355		
	PoTRA rev5	GTGTTTGTAGTATTTACAGG			
Plasmepsin 4	AIRSAMA_Fwd	ACTGACACTGATGATTTAGAACCCATTT	1353	*P. vivax*	
	AIRSAMA_Rev	TGGAGAGATCTTTCCATCCTAAACCT			
	AIRSAMA_Probe	CAGCAGCGTCGAGTTT			
Plasmepsin 4	Ovale_CCRR9V5_Fwd	ACTCTTGGTTATTTGTCTGCACCTT	1353	*P. ovale*	
	Ovale_CCRR9V5_Rev	CTATGTTACCATAAACAGGTTCTAAATCATCTGT			
	Ovale_CCRR9V5_Probe	TCAGTTGCTTCAACAAATTT			
PF3D7_0718800	pf07_0076_Fwd	CGACCCTGATGTTGTTGTTGGA	79	*P. falciparum*	[11]
	pf07_0076_Rev	GGCTTTTTTCCATTTCTGTAGTTAAGATTCA			
	pf07_0076_Probe	CAACAGCTCCAAAATAT			
Plasmepsin 4	Malariae_CCWR2K1_Fwd	TTCAGTCAGGATATGTAAAACAAAATTATTTAGGTAGT	1356	*P. malariae*	
	Malariae_CCWR2K1_Rev	CCTACTTCCCCTTCACCATAAAACA			
	Malariae_CCWR2K1_Probe	TCGTCTAGTTCTATTACGTCATTTTC			

Next, each extracted RDT sample was pre-amplified [12] with species-specific primers (Table 1). The *P. falciparum* primers targeted the PF3D7_0718800 gene, as described previously [13].

Briefly, each reaction was performed with 3 µL of pre-amplified parasite DNA in a 15 µL total reaction volume (2× qPCR Master Mix, 20× primer-probe mix, and PCR-grade water). Amplification and detection were performed under the following conditions: 15-min incubation at 37 °C prior to amplification, initial denaturation for 15 min at 95 °C, then 40 cycles of denaturation for 10 s at 95 °C, and 1 min at 60 °C with data collection.

The specificities of the* P. malariae*, *P. vivax,* and *P. ovale* spp. real-time assays were validated by Sanger sequencing (Macrogen Corp., Rockville, MD) of the amplicon products, both from the synthesized plasmids and the amplification-positive samples identified in the current study.

### *Plasmodium ovale* follow-up

*Plasmodium ovale curtisi* and *Plasmodium ovale wallikeri* were identified among *P. ovale*–positive samples using PoTRA3 and PoTRA5 primers in a nested PCR amplification of the tryptophan rich repeat region as described previously [10]. The PCR mixture consisted of 5 µL of DNA in a final volume of 20 µL (6 µL Phusion high-fidelity master mix [New England Biolabs, Ipswich, MA, USA], 2 µL of 10 pmol each forward and reverse primer, and 5 µL PCR-grade water). Infections with *P. ovale wallikeri* yielded a product of 245 bp, and *P. ovale curtisi* a 317 bp product.

DNA sequences at loci of interest were determined by direct sequencing of the final PCR product using PoTRA5 primers (Table 1). Sequences are available as GenBank accession numbers KX417699–KX417705.

### High-resolution melting assays

Samples positive for *P. ovale* were additionally tested by high-resolution melting to differentiate sub-species using the porbp2 primers, as described previously [2].

## Results

### Validation of *plasmepsin* 4 *P. ovale* spp., *P. malariae*, and *P. vivax* assays

Amplicon products of these assays using control DNA templates and plasmid controls were confirmed to match sequences deposited to GenBank with 100% sequence concordance.

### Overall RDT performance

**Fig. 2:**
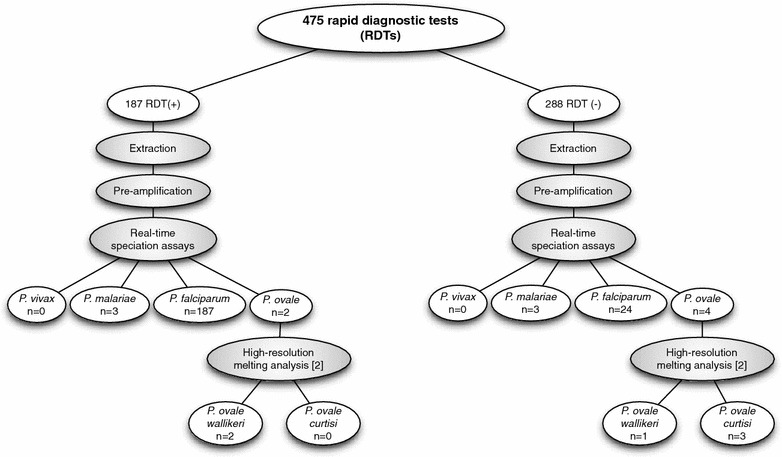
Flow chart of patient samples and species analysis

A total of 475 HRP2-based RDT collected in Kédougou by the *Programme National de Lutte contre le Paludisme* (National Malaria Control Programme or PNLP) were evaluated in this study (Fig. 2). Of these, 187 were positive for *P. falciparum* (RDT positivity rate: 39.3%, 187 of 475 samples). Furthermore, among the 288 samples negative for *P. falciparum* by RDT, 24 were positive for amplification of the *P. falciparum* PF3D7_0718800 gene (Additional file 1: Figure S1), indicating the presence of this species even in RDT-negative samples (PCR positivity rate: 44.4%, 211 of 475 samples). The detection of *Plasmodium* showed similar trends between the two clinic settings (Tomboronkonto and Dindefelo, Additional file 2: Figure S2).

### Evidence of malaria species other than *Plasmodium falciparum* among samples collected by passive case detection in Kédougou, Sénégal

Analysis of the RDT-positive samples revealed co-infection of *P. falciparum* with *P. malariae* (n = 3) and *P. ovale* (n = 2); with one sample positive for all three species. Both *P. ovale* (n = 4) and *P. malariae* (n = 3) were identified from among the 288 RDT-negative samples (Table 1). Although previous reports [7] indicate *P. vivax* infections in this region, in this study population there were no samples positive for *P. vivax.*

### Sympatric circulation of *Plasmodium ovale wallikeri* and *Plasmodium ovale curtisi*

**Fig. 3:**
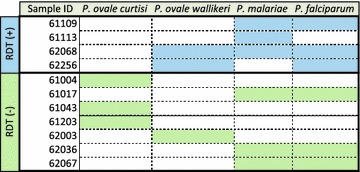
Summary of sample species positivity by rapid diagnostic test status. *Shaded boxes* indicate samples positive for the respective *Plasmodium* species based upon the PCR-based testing described

**Fig. 4:**
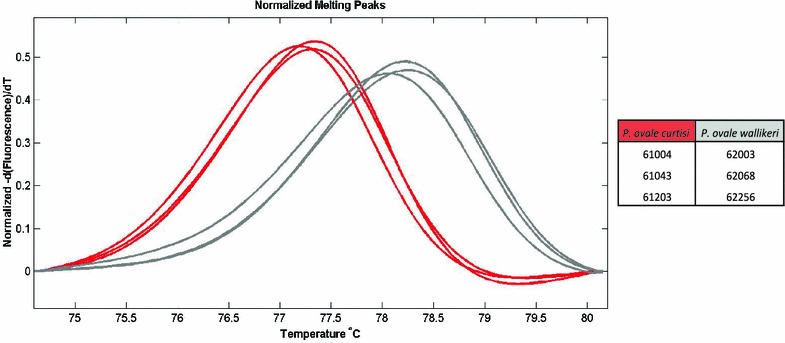
High-resolution melting differentiation of *P. ovale curtisi* and *P. ovale wallikeri*

To further distinguish between the known subtypes of *P. ovale* infection, we employed a high-resolution melting analysis approach and confirmed that two of the RDT-positive samples were positive for *P. ovale wallikeri*; of four RDT-negative samples, three and one were positive for *P. ovale curtisi* and *P. ovale wallikeri*, respectively (Figs. 3, 4). These data indicate that both subtypes of *P. ovale* are evident in the southeastern part of Sénégal.

To confirm the sensitivity and specificity of the genotyping results for *P. malariae* and *P. ovale*, we used a sequencing approach. Sanger sequencing of the positive samples was used to confirm the presence of *P. malariae* and *P. ovale* among the samples, and a BLAST search of the resultant sequences showed high identity with previously reported sequences. Sequencing was also able to distinguish between the *P. ovale curtisi* and *P. ovale wallikeri* identified in these samples; the sequencing results were concordant with those of the high-resolution melt findings.

## Discussion

After significant restructuring in 2005, the Programme National de Lutte contre le Paludisme (PNLP) in Sénégal developed a control strategy implemented between 2006 and 2010 that involved distribution of RDT to all health facilities (2007), nationwide access to artemisinin combination therapies (2007 and 2008), and distribution of insecticide-treated bed nets (2007–2009). While the initial results of this strategy were encouraging based on the nationwide reported incidence of *P. falciparum*, several regions have experienced malaria rebound since 2009 [14]. Of concern is that HRP2-based surveillance may miss both non-*P. falciparum* malaria species as well as low-density *P. falciparum* infections and infections composed exclusively of *P. falciparum* parasites that lack HRP2 and HRP3 expression [15].

The PNLP has also conducted nationwide cross-sectional surveys for disease surveillance. Kédougou, in southeastern Sénégal, is a region that borders Mali and Guinea with relatively high transmission, as indicated by entomological inoculation rates (EIRs) of 100–200 [9, 10]. Recent reports have identified *P. vivax* malaria in Kédougou [7]. Although *P. vivax* was not observed in the population assessed in the current study, *P. ovale wallikeri, P. ovale curtisi,* and *P. malariae* were identified, none of which are tested for by the current RDT. It remains to be determined if this incidence is changing, particularly in comparison with reduced *P. falciparum* transmission in response to increased eradication efforts that target *P. falciparum* vectors and their characteristic behaviors, which might not be common in other *Plasmodium* species. Indeed, there is evidence of significant biological differences between *P. ovale* spp. and *P. falciparum*, with increased latency times reported in patients infected with the former and differences in clinical features between *P. ovale curtisi* and *P. ovale wallikeri* [16]. Additional studies are necessary to more fully characterize the nationwide prevalence of these non-*falciparum* species; to identify their source as importation or local transmission; their primary transmission vectors; and to differentiate between relapse, reinfection, or recrudescence events where applicable.

The results of this study underscore the potential limitations of the routine use of standard RDT for malaria detection in Sénégal both in terms of missing *P. falciparum* infections as well as missing non-*P. falciparum* malaria infections in Kédougou, Sénégal. The current RDT likely detects most of the infections in this region, particularly those without co-infections; however, while the relative proportion of non-*falciparum* infections remains low, it may increase with improved patient access to diagnosis and treatment. Furthermore, the findings of the current study, including the presence of *P. falciparum* genetic material in RDT-negative samples and subsequent differences in positivity rates (39.3 vs. 44.4%), are similar to those of previous reports [17] that PCR-based detection of parasites genomic material is more sensitive than standard RDT. This suggests the need for more sensitive methods to detect infections, particularly in asymptomatic patients and in the context of declining transmission and malaria incidence rates. This study developed tools that allow for the sensitive identification of these infections for surveillance of the incidences of infection and their changes in response to control efforts.

The lack of detection of *P. falciparum* may be due to either low parasite densities, below the level of detection of the RDT or lack of specific HRP2 loci in the parasite population that results in a negative diagnostic test. Reports of non-falciparum malaria are of concern with control and elimination efforts that increasingly rely on HRP2-based and *P. falciparum*-specific RDT, as these may miss infections that contribute to continued malaria transmission, and thus may undermine elimination efforts and impact morbidity and mortality due to malaria. Failure to detect malaria infections other than *P. falciparum* is most likely is due to the specificity of the RDT, which was not designed to detect these malaria species. Regardless of the specific reasons why the RDT failed to detect these infections, these findings point to the need for appropriate diagnosis and surveillance of malaria infection to provide both adequate clinical treatment and to direct elimination strategies to address the nature of ongoing malaria infection in a given endemic setting. Future studies are necessary to assess and track the prevalence of HRP2 deletions in endemic populations in this region.

This study has several limitations. First, the pre-amplification step, like nested and multi-step PCR reactions, offers increased sensitivity to detect parasites at low concentrations, but prevents us from determining the threshold cycles (Ct) values of the original samples; furthermore, RDT were administered by regular health care providers rather than staff specifically trained for malaria testing. Blood smears were not obtained from the patients, and trained microscopists were not available; therefore, parasite densities in these patients were not determined. Finally, the sampling scheme was not a cross-sectional study, so there is a potential bias in the study population. Future cross-sectional studies including larger sample sizes and asymptomatic patients are necessary to validate these findings.

Despite these limitations, the findings of this study suggest the importance of assessing the true prevalence of specific malaria infections using appropriate diagnostic methods to evaluate the requirements for health care services and develop appropriate strategies for malaria elimination. Such quantification is increasingly important in order for malaria elimination programmes to implement species-appropriate detection, prevention, and treatment strategies.

## Additional files

**Additional file 1: Figure S1.** *Plasmodium* spp. detection in Kédougou, 2013–2014 (n = 475 samples).

**Additional file 2: Figure S2.** *Plasmodium falciparum* detection in Kédougou by health center, 2013–2014 (n = 475).
