# Supplementary figures and images for: Evidence of non-Plasmodium falciparum malaria infection in Kédougou, Sénégal
Source: Malar J. 2017 Jan 3;16:9. doi: 10.1186/s12936-016-1661-3 (PMC5209815; doi:10.1186/s12936-016-1661-3)

## *Plasmodium* positivity in Kédougou, 2013-2014

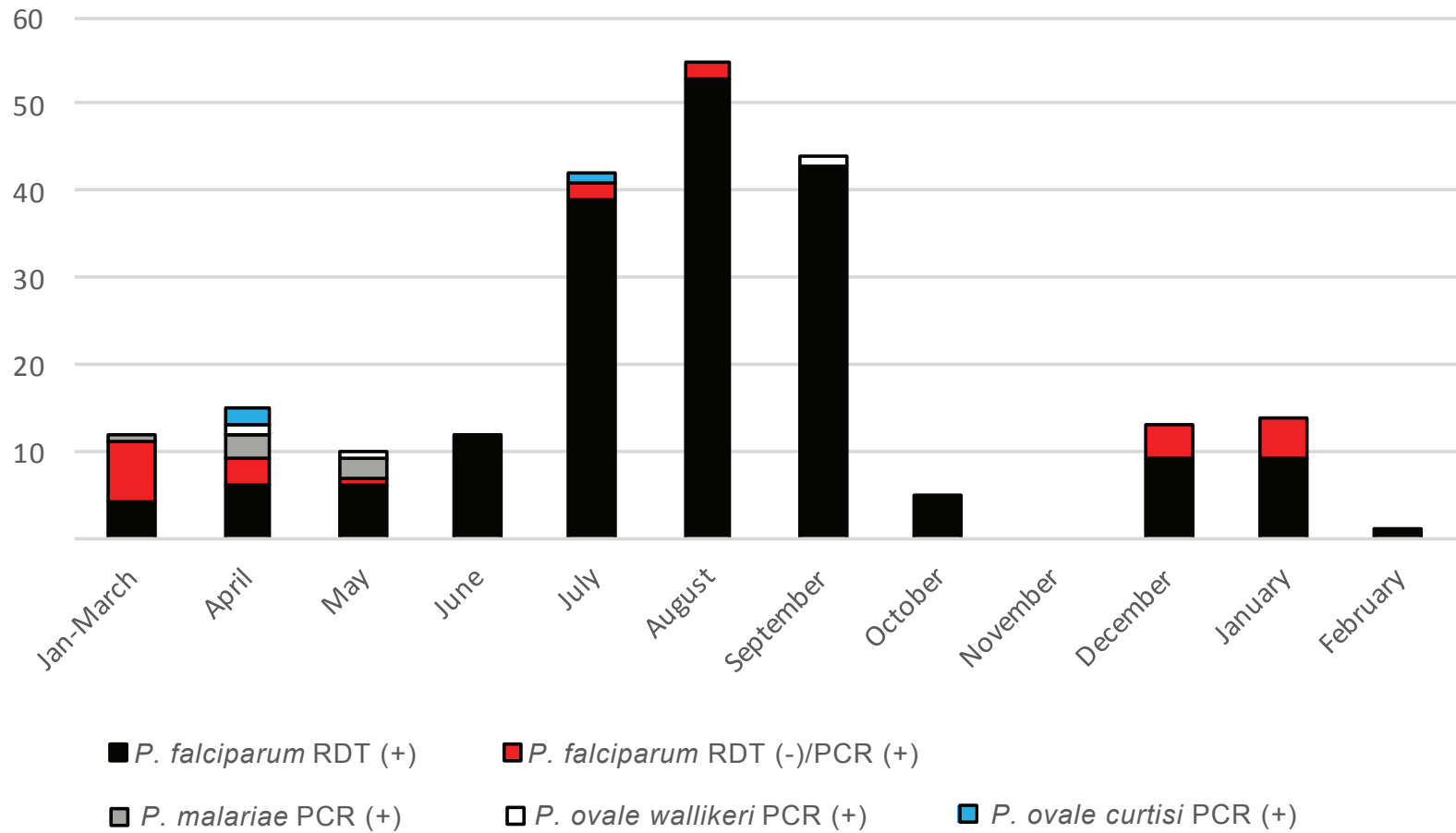

Supplement: Supplementary file 1 — Additional file 1: Figure S1. Plasmodium spp. detection in Kédougou, 2013–2014 (n = 475 samples). [file 12936_2016_1661_MOESM1_ESM.pdf]

*P. falciparum* positivity by clinic in Kédougou, 2013-2014

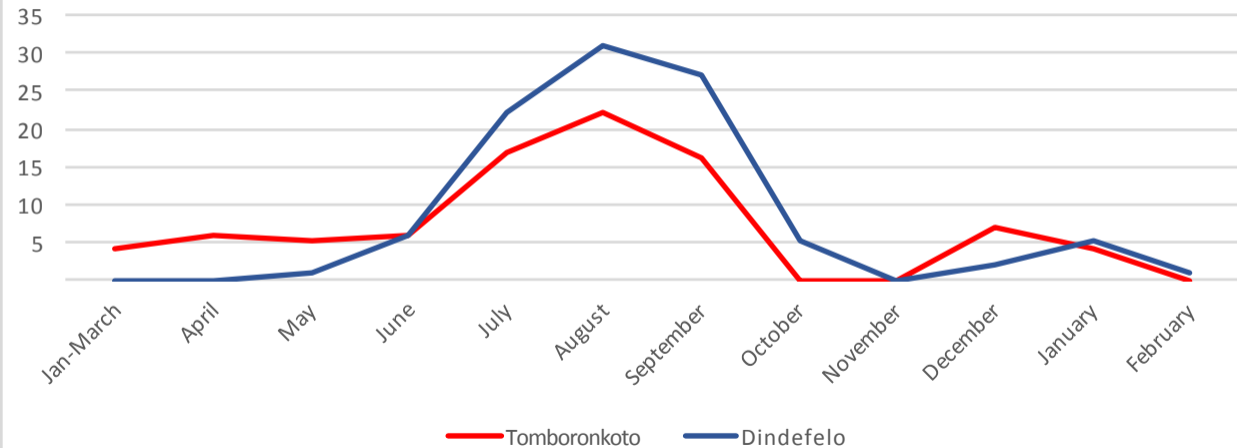

Supplement: Supplementary file 2 — Additional file 2: Figure S2. Plasmodium falciparum detection in Kédougou by health center, 2013–2014 (n = 475). [file 12936_2016_1661_MOESM2_ESM.pdf]
